# Clinical features and outcome of Guillain–Barre syndrome in Saudi Arabia: a multicenter, retrospective study

**DOI:** 10.1186/s12883-021-02314-5

**Published:** 2021-07-12

**Authors:** Mohammed H. Alanazy, Sawsan S. Bakry, Afnan Alqahtani, Norah S. AlAkeel, Naael Alazwary, Afag M. Osman, Rania A. Mustafa, Talal M. Al-Harbi, Sameeh O. Abdulmana, Aimee C. Amper, Yousef Aldughaythir, Abdulrahman S. Ali, Seraj Makkawi, Alaa Maglan, Loujen Alamoudi, Feras Alsulaiman, Majed Alabdali, Aysha A. AlShareef, Ahmad R. Abuzinadah, Ahmed K. Bamaga

**Affiliations:** 1 Division of Neurology, Department of Internal Medicine, King Saud University Medical City and College of Medicine, King Saud University, Riyadh, Saudi Arabia; 2 College of Medicine, King Saud University, Riyadh, Saudi Arabia; 3 Department of Medicine, Security Forces Hospital Program, Riyadh, Saudi Arabia; 4 Department of Neurology, Prince Sultan Military Medical City, Riyadh, Saudi Arabia; 5 Neurology Department, Neuroscience Center, King Fahad Specialist Hospital-Dammam, Dammam, Saudi Arabia; 6 Neurology Department, National Neuroscience Institute, King Fahad Medical City, Riyadh, Saudi Arabia; 7 College of Medicine, King Saud Bin Abdulaziz University for Health Sciences, Jeddah, Saudi Arabia; 8 King Abdullah International Medical Research Center, Jeddah, Saudi Arabia; 9 Department of Medicine, Ministry of the National Guard-Health Affairs, Jeddah, Saudi Arabia; 10 Department of Neurology, King Fahad Hospital of the University, Imam Abdulrhman Bin Faisal University, Dammam, Saudi Arabia; 11 Internal Medicine Department, Neurology Division, Neuromuscular Medicine Unit, Faculty of Medicine and King Abdulaziz University Hospital, King Abdulaziz University, Jeddah, Saudi Arabia; 12 Pediatric department, Neuromuscular Medicine Unit, Faculty of Medicine and King Abdulaziz University Hospital, King Abdulaziz University, Jeddah, Saudi Arabia

**Keywords:** Acute inflammatory demyelinating polyradiculoneuropathy, Acute motor axonal neuropathy, Acute motor-sensory axonal neuropathy, Guillain–Barre Syndrome, Intravenous immunoglobulin, Saudi Arabia

## Abstract

**Background:**

Guillain–Barre syndrome (GBS) is an inflammatory polyradiculoneuropathy characterized by rapidly evolving weakness and areflexia, reaching nadir within 4 weeks. Data on the characteristic of GBS in Saudi Arabia are limited. This study aimed to describe the clinical, electrophysiological, and laboratory characteristics and outcome of a multicenter cohort of patients with GBS.

**Methods:**

This is a retrospective multicenter nationwide study. Patients who had GBS, identified through Brighton Criteria, between January 2015 and December 2019 were included. Data collected included demographics, clinical features, cerebrospinal fluid profile, reported electrophysiological patterns, treatment, and outcome. Reported GBS subtypes were compared using chi-square, Fisher's exact, or Mann–Whitney U tests, as appropriate.

**Results:**

A total of 156 patients with GBS were included (men, 61.5%), with a median age of 38 (interquartile range, 26.25–53.5) years. The most commonly reported antecedent illnesses were upper respiratory tract infection (39.1%) and diarrhea (27.8%). All but two patients (98.7%) had weakness, 64.1% had sensory symptoms, 43.1% had facial diplegia, 33.8% had oropharyngeal weakness, 12.4% had ophthalmoplegia, and 26.3% needed mechanical ventilation. Cytoalbuminological dissociation was observed in 69.1% of the patients. GBS-specific therapy was administered in 96.8% of the patients, of whom 88.1% had intravenous immunoglobulin, and 11.9% had plasmapheresis. Approximately half of the patients were able to walk independently within 9 months after discharge, and a third regained the ability to walk independently thereafter. Death of one patient was caused by septicemia. Acute inflammatory demyelinating polyradiculoneuropathy was the most commonly reported GBS subtype (37.7%), followed by acute motor axonal neuropathy (29.5%), and acute motor-sensory axonal neuropathy (19.2%).

**Conclusion:**

The clinical and laboratory characteristics and outcome of GBS in the Arab population of Saudi Arabia are similar to the international cohorts. The overall prognosis is favorable.

## Background

Guillain–Barre syndrome (GBS) is the most common cause of acute neuropathy worldwide, affecting approximately 1–2 per 100,000 person-years [1]. It has first been described in 1916, and since then, more cases have been recognized, leading to the recognition that GBS comprises a wide spectrum of symptoms with different clinical outcomes [2]. Therefore, it has been categorized into different subtypes based on clinical, pathophysiological, and electrophysiological findings. The most common GBS subtypes include acute inflammatory demyelinating polyradiculoneuropathy (AIDP), acute motor axonal neuropathy (AMAN), and acute motor-sensory axonal neuropathy (AMSAN) [3]. Such classification has a diagnostic and prognostic value with AIDP variant having a favorable prognosis compared to the other two subtypes [4].

Previous studies have revealed regional variations in the prevalence of different subtypes of GBS [5]. In North America and Europe, AIDP has been reported as the predominant subtype [5], whereas the axonal subtype constituted only a small percentage of GBS cases. The contribution of the axonal subtype is higher in Northern China, Japan, and South America [6]. Data on GBS from Arab countries are limited [7]. One study in Kuwait analyzed 41 patients with GBS and found AIDP to be the most common subtype [8]. Another study from Iran reported AMSAN to be the predominant subtype among its local study population [9]. A single-center study conducted in Saudi Arabia did not provide data on GBS subtypes [10]. It is essential to determine the regional prevalence of GBS subtypes and understand their pathophysiology, which may eventually aid in developing a GBS subtype-targeted immune therapy.

This nationwide multicenter retrospective study aimed to highlight the clinical, laboratory, and electrophysiological characteristics and outcome in adult patients with GBS in Saudi Arabia and compare these parameters between GBS subtypes and our data to the international studies.

## Methods

### Study design

This is a retrospective multicenter study that included patients diagnosed with GBS between January 2015 and December 2019. Eight tertiary centers across the country participated in the study: four from the Central region (Riyadh), two from the Eastern region (Khobar and Dammam), and two from the Western region (Jeddah). The study was approved by the institutional review board at King Saud University and all other participating centers (Security Forces Hospital, Prince Sultan Military Medical City, King Fahad Medical City, King Fahad Specialist Hospital-Dammam, King Fahad Hospital of the University, King Saud Bin Abdulaziz University for Health Sciences, King Abdulaziz University). The need for informed consent was waived by all the participating centers.

### Study population

Patients aged 18 years or more with GBS were identified through the electronic health system and/or electrophysiology database in each center. The inclusion criteria were adopted from the previously validated Brighton criteria, [11, 12] and included (1) acute or subacute flaccid weakness involving lower and/or upper limbs; (2) monophasic disease, reaching nadir of weakness between 12 h and 4 weeks; and at least one of the following: (a) hyporeflexia or areflexia in the weak limbs, (b) cytoalbuminological dissociation defined as the combination of cerebrospinal fluid (CSF) protein level > 0.45 g/L and cell count < 50 cells/µl, and (c) the reported electrophysiological features are compatible with a subtype of GBS. We accepted the subtype classification documented by the treating neurologists/neuromuscular specialists, because the lack of availability of electrophysiological raw data in most centers hindered our ability to confirm subtype classification. We allowed relative preservation of deep tendon reflexes in otherwise typical GBS presentations as has been described in a minority of patients [12–14]. We included patients who presented with the classical triads of Miller-Fisher syndrome (MFS): ataxia, ophthalmoplegia, and areflexia. The exclusion criteria were (1) progressive weakness for > 8 weeks, (2) CSF cell count ≥ 50 cells/µl, (3) onset of post-bariatric surgery, and (4) symptoms explained by an alternative diagnosis.

### Study variables

In addition to demographic variables, we collected the following GBS variables: antecedent event type and time before onset, clinical features, time of symptom onset, forced vital capacity (FVC) at admission, reported electrophysiological classification (AIDP, AMAN, AMSAN, equivocal, and inexcitable), radiological features, CSF profile, therapy received, admission to an intensive care unit (ICU), mechanical ventilation, tracheostomy, duration of ICU stay, duration of hospitalization, and ability to walk at follow-up.

### Analysis

Continuous variables were presented as median and interquartile range (IQR). Nominal variables were presented as counts and proportions. A chi-square test or Fisher's exact test was used to assess differences in binomial variables between GBS subtypes, as appropriate. A Mann–Whitney U test was used to assess differences in continuous variables between GBS subtypes. A two-sided *P*-value < 0.05 was considered statistically significant. For multiple comparisons, a Bonferroni-corrected *P*-value was calculated by multiplying the *P*-value with the number of comparisons conducted. Data were analyzed using the software SPSS (version 23, Chicago, IL).

### Results

Initial screening revealed 169 patients, of whom 13 were excluded for the reason of not meeting the inclusion criteria or due to the presence of a mimicking disease and 156 patients were included in the study (96 [61.5%] were men, and 60 [38.5%] were women). Demographic and clinical characteristics of the patients with GBS are shown in Table 1. The median age was 38 (IQR 26.25–53.5) years. Approximately, half of the patients (51.9%) were aged 18–39 years (57 men, 24 women), 29.5% were aged 40–59 years (24 men, 22 women), and 18.6% were aged ≥ 60 years (15 men, 14 women).

**Table 1:** Demographic and clinical characteristics of patients with GBS at the time of admission

Variable	N (%) or median (IQR)
Age, years	38 (26.25–53.5)
Male: female	1.6: 1
Antecedent illness^a^	101 /133 (75.9)
URTI	52/133 (39.1)
Diarrhea	37/133 (27.8)
Surgery	6/133 (4.5)
Others^b^	6/133 (4.5)
Seasonal distribution	
Winter	36/119 (30.3)
Spring	32/119 (26.9)
Summer	19/119 (16.0)
Autumn	32/119 (26.9)
Clinical features at admission^c^	
Weakness	154/156 (98.7)
Sensory disturbance	100/156 (64.1)
Facial diplegia	66/153 (43.1)
Oropharyngeal weakness	52/154 (33.8)
Ophthalmoplegia	19/153 (12.4)
Dyspnea	40/154 (26.0)
Ataxia	2/154 (1.3)
Areflexia or hyporeflexia	133/149 (89.3)
Sensory (small fiber) involvement	55/134 (41.0)
Sensory (large fiber) involvement	23/117 (19.7)
Urinary retention	2/156 (1.3)
Autonomic instability	11/156 (7.1)
Able to walk independently at admission	56/151 (37.1)
ICU admission^d^	52/156 (33.3)
Mechanical ventilation	41/156 (26.3)
Tracheostomy	25/156 (16.0)

^a^Median time before onset = 12 (7–14) days

^b^2 had fever; 1 had pneumonia; 1 had pyelonephritis; 1 had diabetic ketoacidosis; 1 was postpartum

^c^Median time from onset = 6 (3–12) days

^d^Duration of ICU stay = 17.5 (7.5–34.5) days

### Clinical features

Approximately three-fourths of the patients had an antecedent event, with upper respiratory tract infection (URTI) being the most common antecedent infection, followed by diarrhea (Table 1). All but two patients (98.7%) had weakness at admission, 64.1% had sensory symptoms, 43.1% had facial diplegia, 33.8% had oropharyngeal weakness, and 12.4% had ophthalmoplegia. The GBS clinical presentation was the classic sensorimotor form in 100 (64.1%) patients. Fifty-four patients (34.6%) had a pure motor form, and 2 patients (1.3%) had MFS. Approximately, one-fourth of the patients needed mechanical ventilation, and 16.0% needed tracheostomy. The median duration of ICU stay was 17.5 (7.5–34.5) days.

### Investigations and electrophysiological subtypes

CSF analysis was performed in 123 (78.8%) patients, at a median of 5 (IQR, 2–14) days after symptom onset, and “cytoalbuminological dissociation” was observed in 85 (69.1%) patients.

(Table 2). CSF protein concentration was increased in 44 (59.5%) patients at < 8 days, 19 (82.6%) patients at 8 – 14 days, and 22 (84.6%) patients at > 14 days after disease onset. FVC was available from 84 (53.8%) patients at admission. Less than a third of these patients had a low FVC, defined as FVC < 1.5 L or 20 mL/kg. Nerve root enhancement was observed in 32 (42.7%) of the 75 patients who had spinal cord magnetic resonance imaging. Nerve conduction study (NCS) reports were available from 146 (93.6%) patients, and the procedure was performed at a median of 10 (IQR 5–20) days after symptom onset. Of these patients, 55 (37.7%) were considered to have AIDP, 43 (29.5%) were considered to have AMAN, 28 (19.2%) were considered to have AMSAN, and 9 (6.2%) were considered as equivocal, Table 2.

**Table 2:** Laboratory and electrophysiological features, therapy, and outcome of patients with GBS

Variable	N (%) or median (IQR)
CSF ^a^	123 (78.8)
Protein level, g/L	0.63 (0.39–1.13)
Protein > 0.45 g/L	85 (69.1)
Cell count/µl	1.0 (0–3)
Cell count > 5/µl	13 (10.6)
Cytoalbuminological dissociation	85 (69.1)
Low FVC (< 1.5L or 20 mL/kg) at admission	23/84 (27.4)
MRI spine: root enhancement	32/75 (42.7)
MRI brain: cranial nerve enhancement	2/76 (2.6)
Electrophysiological subtypes as reported^b^	146 (93.6)
AIDP	55/146 (37.7)
AMAN	43/146 (29.5)
AMSAN	28/146 (19.2)
Equivocal	9/146 (6.2)
Normal	11/146 (7.5)
Received GBS Therapy^c^	151 (96.8)
IVIg	133/151 (88.1)
PLEX	18/151 (11.9)
Repeated therapy ^d^	38/151 (25.2)
IVIg–PLEX	19/38 (50.0)
IVIg–IVIg	11/38 (28.9)
PLEX–IVIg	7/38 (18.4)
PLEX–PLEX	1/38 (2.6)
Duration of hospitalization (*N* = 145, 93%)	2.4 (1–8) weeks
Outcome at follow-up	97 (62.2%)
Able to walk independently at < 6 months	37/97 (38.1)
Able to walk independently at 6 – 9 months	11/97 (11.3)
Able to walk independently at > 9 months	32/97 (33.0)
Able to walk with support at > 9 months	10/97 (10.3)
Unable to walk at > 9 months	7/97 (7.2)
Died (Septicemia)	1/156 (0.64)

*AIDP* acute inflammatory demyelinating polyradiculoneuropathy, *AMAN* acute motor axonal neuropathy, *AMSAN* acute motor-sensory axonal neuropathy, *IVIg* intravenous immunoglobulin, *MFS* Miller-Fisher syndrome, *N* number of patients for whom data are available, *PLEX* plasma exchange

^a^Median time after the onset of symptoms = 5 (2–14) days, and maximum CSF cell count = 23 cells/µl

^b^Median time after the onset of symptoms = 10 (5–20) days

^c^Median time after the onset of symptoms = 7 (4–13.75) days

^d^Median time after first therapy = 17 (8.5–28.5) days

### Therapy and outcome

GBS-specific therapy was administered in 151 (96.8%) patients at a median duration of 7 (IQR, 4–13.75) days after symptom onset (Table 2). The majority of these patients (88.1%) had intravenous immunoglobulin (IVIg), and the remainder (11.9%) had plasmapheresis. A quarter of these patients had a second course of therapy at a median duration of 17 (IQR, 8.5–28.5) days after completion of the first therapy. The median duration of hospitalization for the 145 (93%) patients with available data was 2.4 (1–8) weeks. Ability to walk was reported at follow-up in 97 (62.2%) patients. Approximately half of these patients were able to walk independently within 9 months after discharge, and a third regained the ability to walk independently thereafter. Death of one patient was caused by septicemia.

### Comparison between GBS subtypes

Comparisons between GBS subtypes are shown in Tables 3 and 4. There were no significant differences in the clinical features between the GBS subtypes with the exception that there was a higher proportion of patients who were able to walk independently at the time of admission in the AIDP subtype compared to that in the axonal subtype (combined AMAN and AMSAN). Patients with AMSAN were significantly more likely to have oropharyngeal weakness than those with AMAN. An antecedent URTI was more frequent in patients with AIDP compared to those with the axonal subtype, and diarrhea was more frequent in patients with AMAN compared to those with AMSAN. CSF protein level was significantly lower in patients with AMAN (median, 0.5 g/L; IQR, 0.32–0.86) than in patients with AIDP (median, 0.8 g/L; IQR, 0.51–1.4; Bonferroni-corrected *P* = 0.05) and AMSAN (median 0.93 g/L; IQR, 0.59–2.4; Bonferroni-corrected *P* = 0.036).

**Table 3:** Clinical and laboratory characteristics and outcome comparing axonal versus demyelinating subtypes of GBS

Variable	Axonal (AMAN and AMSAN), (*N* = 71)	Demyelinating (AIDP) (*N* = 55)	*P*-value	OR (95% CI)
	N (%) or Median (IQR)	N (%) or Median (IQR)		
Age, years	38 (26–54)	39 (28–50)	0.88	
Sex (men)	40/71 (56.3)	36/55 (65.6)	0.30	
Time of antecedent event before symptom onset, days	10 (7–14)	14 (7–21)	0.14	
Time from symptom onset to presentation, days	6 (2–13)	7 (4–14)	**0.043**	
Clinical features at admission				
Ophthalmoplegia	3/68 (4.4)	3/55 (5.5)	1.0	
Facial diplegia	27/68 (39.7)	28/55 (50.9)	0.21	
Oropharyngeal weakness	24/69 (34.8)	17/55 (30.9)	0.65	
Able to walk independently	16/68 (23.5)	25/53 (47.2)	**0.006**	2.9 (1.3–6.3)
Antecedent infection				
URTI	15/64 (23.4)	27/50 (54.0)	**0.001**	3.8 (1.7–8.6)
Diarrhea	27/64 (42.2)	9/50 (18.0)	**0.006**	3.3 (1.4–8.0)
Investigations				
CSF protein level, g/L	0.65 (0.39–1.1)	0.8 (0.51–1.4)	0.27*	
CSF protein > 0.45 g/L	38/57 (66.7)	35/44 (79.5)	0.15	
Cell count > 5/µl	3/57 (5.3)	4/44 (9.1)	0.70	
Low FVC	13/39 (33.3)	9/34 (26.5)	0.52	
Nerve root enhancement on MRI	16/52 (30.8)	10/34 (29.4)	0.89	
Hospital course				
Repeated therapy	23/68 (33.8)	8/53 (15.1)	**0.019**	2.9 (1.2–7.1)
ICU admission	30/71 (42.3)	12/54 (22.2)	**0.019**	2.6 (1.2–5.7)
Mechanical ventilation	26/58 (44.8)	8/48 (16.7)	**0.002**	4.1 (1.6–10.2)
Tracheostomy	17/56 (30.4)	3/47 (6.4)	**0.002**	6.4 (1.7–23.5)
Duration of ICU stay, days	28 (15–70)	12 (7–19.5)	0.10	
Duration of hospitalization, weeks	4 (1.4–13.8)	2 (1–4)	**0.003**	
Outcome				
Able to walk independently at follow-up	38/53 (71.7)	38/43 (88.4)	**0.045**	3 (1.0–9.1)
Duration of follow-up, months	11 (4.6–17.4)	5 (2–12)	**0.03**	

^*^CSF protein level was higher in AIDP than in AMAN (median, 0.5; IQR, 0.32–0.86), Bonferroni-corrected *P* = 0.05

**Table 4:** Clinical and laboratory characteristics and outcome comparing AMAN versus AMSAN subtypes of GBS

Variable	AMAN (*N* = 43)	AMSAN (*N* = 28)	*P*-value		OR (95% CI)
	N (%) or Median (IQR)	N (%) or Median (IQR)			
Age, years	38 (25–52)	38 (27.25–60.25)	0.34		
Sex (men)	25/43 (58.1)	15/28 (53.6)	0.70		
Time of antecedent event before symptom onset, days	10 (7–14)	10 (5–14)	0.98		
Time from symptom onset to presentation, days	4 (2–7)	7 (2–14)	0.07		
Clinical features at admission					
Ophthalmoplegia	1/43 (2.3)	2/25 (8.0)	0.55		
Facial diplegia	14/43 (32.6)	13/25 (52.0)	0.11		
Oropharyngeal weakness	9/43 (20.9)	15/26 (57.7)	**0.002**	5.2 (1.8–15)	
Able to walk independently	11/41 (26.8)	5/27 (18.5)	0.43		
Antecedent infection					
URTI	7/38 (18.4)	8/26 (30.8)	0.25		
Diarrhea	22/38 (57.9)	5/26 (19.2)	**0.002**	5.8 (1.8–18.6)	
Investigations					
CSF Protein level, g/L	0.5 (0.32–0.86)	0.93 (0.59–2.4)	0.036*		
CSF protein > 0.45 g/L	20/36 (55.6)	18/21 (85.7)	**0.02**	4.8 (1.2–19.2)	
Cell count > 5/µl	3/36 (8.3)	0/21 (0.0)	0.30		
Low FVC at admission	7/26 (26.9)	6/13 (46.2)	0.29		
Nerve root enhancement on MRI	10/33 (30.3)	6/19 (31.6)	0.94		
Hospital course					
Repeated therapy	10/41 (24.4)	13/27 (48.1)	**0.043**	2.9 (1.02–8.1)	
ICU admission	10/43 (23.3)	20/28 (71.4)	** < 0.001**	8.3 (2.8–24.4)	
Mechanical ventilation	9/31 (29.0)	17/27 (63.0)	**0.01**	4.2 (1.4–12.5)	
Tracheostomy	5/29 (17.2)	12/27 (44.4)	**0.03**	3.8 (1.1–13.1)	
Duration of ICU stay, days	16 (5–29.25)	33 (20–81)	**0.049**		
Duration of hospitalization, weeks	2 (1–8.5)	12 (3–44)	**0.001**		
Outcome					
Able to walk independently at follow-up	27/34 (79.4)	11/19 (57.9)	0.10		
Duration of follow-up, months	12.5 (4.6–20.8)	8 (3.5–15.2)	0.23		

^*^Bonferroni-corrected

Regarding hospital course, there were more frequent repeated therapy, ICU admission, mechanical ventilation, and tracheostomy and longer duration of hospitalization in patients with the axonal subtype compared to those with AIDP, Table 3. These variables and duration of ICU stay were also more frequent in patients with AMSAN compared to those in patients with AMAN, Table 4. Despite the shorter follow-up period in patients with AIDP, a higher proportion of these patients were able to walk independently at follow-up compared to those with the axonal subtype.

## Discussion

This is the first nationwide multicenter study on adult patients with GBS in Saudi Arabia. The earliest studies included a small number of patients recruited from a single center [10, 15, 16]. A previous local study in the 1990s [10] showed that GBS is more predominant in men than women in the third and fourth decades. In contrast, there has been a remarkable attenuation in men to women ratio whereby it decreased from the 9:1 ratio in a previous local study [10] to 2.4:1 in this study. The predominance of GBS in men is consistent with those in previous regional and international studies [5, 8, 14, 17–22]. A meta-analysis revealed that the incidence of GBS increases with age [23], and this has also been reported in the international GBS outcome study (IGOS) that included 925 patients worldwide [5]. In contrast, only 18.6% of our patients were in the older age group. This discrepancy in the age distribution between our cohort and international studies may be explained by variations in the demography of the general populations. In the 2019 census, only 5.5% of the Saudi population was 60 years and older [24], reflecting a lower number of persons at risk in this age category compared to the younger age categories.

Literature review revealed that two-thirds of patients with GBS have an antecedent respiratory or gastrointestinal tract infection in the 6 weeks preceding the onset of GBS [1]. In our study, 66.9% of the patients had an URTI or diarrhea with a median time of 12 days before symptom onset. Compared to other GBS subtypes, we observed an association between antecedent URTI and AIDP and between antecedent diarrhea and AMAN, which is consistent with data from previous studies [14, 22, 25].

Although the diagnosis of GBS is based on the clinical criteria, CSF examination is required to substantiate the diagnosis and exclude competing diagnoses such as infectious or neoplastic polyradiculitis. The typical CSF finding in GBS is cytoalbuminological dissociation [11]. Concurring with previous studies [5, 12, 26], CSF protein level was highly dependent on the timing of lumbar puncture. The proportion of patients with an elevated CSF protein level was lower in the first week compared to that in the second and third week after symptom onset. Of all patients, 69.1% had an elevated CSF protein level. This was higher than that reported in the Asian cohort (56%) [26], and similar to that reported in the Dutch (64%) and IGOS (68%) cohorts [5, 12]. The proportion of patients with mild pleocytosis, defined as a CSF cell count of 5 – 50 cells/µl was lower in this study (10.6%) than that reported in the Asian (26%), Dutch (15%) and IGOS (19%) cohorts [5, 12, 26]. Because no patient had a CSF cell count > 50 cells/µl, “cytoalbuminological dissociation” was determined by elevated CSF protein level, and observed in 69.1% of the patients in this study, compared to 55% in the Asian cohort [26], 64% in the Dutch cohort [12], and 67% in the IGOS cohort [5]. The difference in CSF protein levels between this study and the Asian study is not explained by the timing of lumbar puncture because similar proportions of patients in both studies had lumbar puncture in the first (60%) and second week (20%) after symptom onset. It is possible that a variability in the proportions of GBS subtypes could have contributed to this discrepancy, although this hypothesis cannot be evaluated because GBS subtypes were not provided in the Asian cohort. For example, we observed a higher CSF protein level in patients with AIDP and AMSAN compared to that in patients with AMAN. This supports the findings of a recent study that reported a higher CSF protein in the sensorimotor and demyelinating subtypes compared to the pure motor and axonal subtypes [27]. The relatively lower CSF protein in patients with AMAN may be related, in part, to the selective targeting of the nodal/paranodal region, as opposed to the involvement of sensory and motor nerve roots in AIDP [6].

In this study, the AIDP was the most commonly reported electrophysiological subtype, accounting for 37.7% of the GBS cases. The second and third prevalent subtypes were AMAN and AMSAN, accounting for 29.5% and 19.2% of the cases, respectively. The corresponding proportions in a cohort of 44 patients from Oman were 52% for AIDP, 30% for AMAN, and 14% for AMSAN [17]. These two timely studies from the Arabian Peninsula showed similar predominance of the GBS subtypes, although the proportions of patients with AIDP were relatively lower in this study compared to those in the Omani study. A relatively older study from Kuwait reported a higher proportion of AIDP (68%) and a lower proportion of the axonal subtype (15%) [8]. Two recent retrospective studies from Northern and Southern China reported different frequencies of the electrophysiological subtypes of GBS. In the former study, AMAN was the predominant subtype (55.8%) and AIDP occurred less frequently (21.2%) [22], whereas the study from Southern China reported a relatively higher frequency of AIDP (49.0%) compared to a lower frequency of AMAN (18.8%) [28]. The proportions of GBS subtypes in this study are relatively comparable to data from Southern China [28]. However, the frequency of AMAN in China and Saudi Arabia is still higher than that reported from North America and Europe (3.0%) [3]. The geographic discrepancies in the incidence of AMAN and AIDP may be influenced by environmental factors, differences in the frequencies and types of preceding infections, and genetic polymorphisms of *Campylobacter jejuni* strains. The relative similarity between our data and data from Southern China [28], representing two different ethnic groups, argues against a role for human genetic polymorphisms in influencing GBS subtype.

Overall, despite the prolonged recovery time and residual weakness in some patients, the outcome in our cohort is favorable. The ability to walk independently within 6 months was achieved in a lower proportion of patients in our cohort (38.1%) compared to that at 6 months in the IGOS cohort (77%). This discrepancy was due to the retrospective nature of our study wherein follow-up visits were not conducted at fixed time points. Nonetheless, the proportions of patients who were able to walk independently were very similar when comparing our data at > 9 months (82.5%) to the IGOS cohort at 12 months (81%) [5]. In agreement with the literature [5], our data showed that the ability to walk independently at follow-up was achieved in a higher proportion of patients with AIDP than those with the axonal subtype.

The need for mechanical ventilation has been reported in 20%–30% of patients with GBS [12]. Likewise, approximately one-fourth of our patients required mechanical ventilation. Contradicting the report by Durand et al., who observed that the risk of requiring mechanical ventilation was higher in patients with demyelinating neuropathy [29], we observed that a high proportion of patients with the axonal subtype, particularly AMSAN, required mechanical ventilation and subsequent tracheostomy. This was reflected on a longer duration of hospitalization in the axonal subtype compared to the AIDP. Among the patients with axonal subtype, a higher proportion of patients with AMSAN needed mechanical ventilation and had longer duration of ICU stay and hospitalization than those with AMAN.

## Limitations

The inherent limitations of retrospective studies are to be considered when interpreting the findings of this study, including the bias introduced by missing data, lack of standard assessment, and differences in timing of follow-up. The retrospective and multicentric nature of the study and the lack of availability of NCS raw data hindered our ability to independently confirm the GBS subtypes documented by the treating neurologist at each hospital. Antiganglioside antibodies were not included because these were not tested in almost all centers.

## Conclusions

The clinical and laboratory characteristics of patients with GBS in Saudi Arabia and their outcome are similar to those in the international cohorts. The reported GBS subtypes in order of frequency are AIDP, AMAN, and AMSAN. A prospective study with more rigorous data collection will aid in evaluating the incidence and burden of the disease nationwide.

## Data Availability

Data and relevant material is available upon request. However, access to the data is restricted to the public and require appropriate approvals from the authorized and participating parties.

## Abbreviations

AIDP: Acute inflammatory demyelinating polyradiculoneuropathy
AMAN: Acute motor axonal neuropathy
AMSAN: Acute motor-sensory axonal neuropathy
GBS: Guillain–Barre syndrome
ICU: Intensive care unit
IGOS: International Guillain–Barre ´Syndrome Outcome Study
IVIg: Intravenous immunoglobulin
MFS: Miller-Fisher syndrome
NCS: Nerve conduction studies
PLEX: Plasmapheresis
